# Correlating changes in lung function with patient outcomes in chronic obstructive pulmonary disease: a pooled analysis

**DOI:** 10.1186/1465-9921-12-161

**Published:** 2011-12-29

**Authors:** Paul W Jones, James F Donohue, Jerry Nedelman, Steve Pascoe, Gregory Pinault, Cheryl Lassen

**Affiliations:** 1 Division of Clinical Science, St George's, University of London, London, UK; 2 Division of Pulmonary & Critical Care Medicine, University of North Carolina, School of Medicine, Chapel Hill, NC, USA; 3 Novartis Pharmaceuticals Corporation, East Hanover, USA; 4 Novartis Pharma AG, Basel, Switzerland; 5 Novartis Horsham Research Centre, Horsham, West Sussex, UK

**Keywords:** COPD, spirometry, FEV_1_, health status, dyspnoea

## Abstract

**Background:**

Relationships between improvements in lung function and other clinical outcomes in chronic obstructive pulmonary disease (COPD) are not documented extensively. We examined whether changes in trough forced expiratory volume in 1 second (FEV_1_) are correlated with changes in patient-reported outcomes.

**Methods:**

Pooled data from three indacaterol studies (n = 3313) were analysed. Means and responder rates for outcomes including change from baseline in Transition Dyspnoea Index (TDI), St. George's Respiratory Questionnaire (SGRQ) scores (at 12, 26 and 52 weeks), and COPD exacerbation frequency (rate/year) were tabulated across categories of ΔFEV_1_. Also, generalised linear modelling was performed adjusting for covariates such as baseline severity and inhaled corticosteroid use.

**Results:**

With increasing positive ΔFEV_1_, TDI and ΔSGRQ improved at all timepoints, exacerbation rate over the study duration declined (P < 0.001). Individual-level correlations were 0.03-0.18, but cohort-level correlations were 0.79-0.95. At 26 weeks, a 100 ml increase in FEV_1 _was associated with improved TDI (0.46 units), ΔSGRQ (1.3-1.9 points) and exacerbation rate (12% decrease). Overall, adjustments for baseline covariates had little impact on the relationship between ΔFEV_1 _and outcomes.

**Conclusions:**

These results suggest that larger improvements in FEV_1 _are likely to be associated with larger patient-reported benefits across a range of clinical outcomes.

**Trial Registration:**

ClinicalTrials.gov NCT00393458, NCT00463567, and NCT00624286

## Introduction

In the absence of other widely accepted and validated markers for chronic obstructive pulmonary disease (COPD), lung function measurement, specifically forced expiratory volume in 1 second (FEV_1_), has been used as a global marker for pathophysiological changes [1] and by regulators in the drug approval process. Consequently, clinical trials for new products in COPD are typically powered to demonstrate significant improvements in FEV_1_. However, healthcare professionals are more likely to be interested in improvements in patient-reported outcomes such as symptoms and health status, which may better reflect treatment impact on the patient. Decision-makers also require evidence to assess trends across large cohorts of patients.

Several studies have demonstrated a significant relationship between poor lung function and worsened health and economic outcomes in patients with COPD [2-13], but few have investigated whether changes in lung function associated with an intervention are correlated with changes in such endpoints [12-15]. There is good evidence that declining lung function leads to worsened patient outcomes, but a surprising lack of evidence that improvements in lung function are correlated with improvements in symptomatic outcomes.

Indacaterol is a novel, inhaled, ultra-long-acting β_2_-agonist. Initial Phase III trials included over 3000 patients, providing a large pooled dataset. We analysed this dataset in order to examine the relationships between change in FEV_1 _and outcomes including dyspnoea, health status, exacerbations and rescue medication use.

## Methods

### Study design and treatments

This investigation was a pooled analysis of patient-level data from three Phase III, randomised studies: Study 1 (INVOLVE [INdacaterol: Value in COPD: Longer term Validation of Efficacy and safety]) was a double-blind comparison of indacaterol 300 μg or 600 μg once daily with formoterol 12 μg twice daily and placebo for 52 weeks; Study 2 (INHANCE [INdacaterol versus tiotropium to Help Achieve New COPD treatment Excellence]) compared double-blind indacaterol 150 μg or 300 μg once daily with placebo and open-label tiotropium 18 μg once daily for 26 weeks; Study 3 (INLIGHT 1 [INdacaterol: efficacy evaLuation usInG 150 μg doses witH COPD PatienTs]) was a 12-week study comparing double-blind indacaterol 150 μg once daily with placebo for 12 weeks. Patients were permitted to continue inhaled corticosteroid (ICS) monotherapy if the dose and regimen were stable for 1 month before screening, and were to remain stable throughout the study; patients were also permitted rescue salbutamol as needed. Full details have been reported elsewhere [16-18]. All studies were conducted in accordance with the Declaration of Helsinki (1989) and local applicable laws and regulations. Approval was obtained from the Institutional Review Board or Independent Ethics Committee of each participating study centre. All patients provided written informed consent prior to participating in each study included in the pooled analysis. All patient data was anonymised.

### Patients

Patients were male or female, aged ≥ 40 years, with a smoking history of ≥ 20 pack years and a diagnosis of moderate-to-severe COPD [19]. All patients in whom trough FEV_1 _measurements were available both at baseline and at 12 weeks were included. Patients with extreme changes from baseline in trough FEV_1 _(> +500 or < -500 ml) were excluded, as these values were considered erroneous.

### Endpoints

The primary endpoint in all three studies was trough FEV_1 _(average of the 23 h 10 min and 23 h 45 min post-dose value) after 12 weeks of treatment. Trough FEV_1 _at baseline was defined as the average of the FEV_1 _values 50 and 15 min prior to the first dose of study drug. Trough FEV_1 _was assessed at the end of Weeks 4, 8 and 12 in all studies, Weeks 16, 20, 24, 28, 36, 44 and 52 in Study 1, and Weeks 16, 21 and 26 in Study 2. In all studies, spirometry equipment and performance of spirometric testing was required to be in accordance with ATS/ERS standards [20].

Secondary endpoints included health status (using the St George's Respiratory Questionnaire [SGRQ] [21]), and dyspnoea (using the Transition Dyspnoea Index [TDI] [22]; Studies 1 and 2 only). The SGRQ provides scores between 0 and 100, with higher values indicating greater impairment. The TDI is inherently a change from baseline and provides values between -9 and +9, with positive values indicating improvement. Rescue medication use (number of puffs of salbutamol) was recorded by patients in diaries. COPD exacerbations were defined as the onset or worsening of > 1 respiratory symptom for > 3 consecutive days, requiring intensified treatment (e.g. systemic steroids, antibiotics, oxygen) and/or hospitalisation or emergency room visit. Severe exacerbations were those requiring hospitalisation.

### Statistical methods

The primary objective was to examine relationships between patient-reported outcomes and change from baseline in trough FEV_1 _(ΔFEV_1_) using data summarisation and model-based analysis. Outcome variables for both analysis approaches were TDI, change from baseline in SGRQ (ΔSGRQ), rescue medication use and exacerbation rates.

For TDI and ΔSGRQ, relationships were examined with the average of each patient's ΔFEV_1 _through the corresponding week of observation. For rescue medication use and exacerbations, the average ΔFEV_1 _over time on treatment was used.

#### Data summaries and related inferences

TDI and ΔSGRQ were handled as outcome variables at 12, 24/26 and 52 weeks. Responders were patients who achieved at least the minimal clinically important difference (MCID) from baseline (one and four units for TDI and SGRQ, respectively [21,22]). Daily rescue medication use was the number of puffs during treatment divided by the number of days on treatment. Rate of exacerbations was the number of exacerbations on treatment, normalised to 1 year (365 × number of exacerbations while on treatment/days on treatment).

For each of the timepoints, outcomes and responder rates for ΔSGRQ and TDI were tabulated across five categories of ΔFEV_1 _that were chosen to distribute patients approximately equally across categories, and bounded above and below by ± 500 ml. The hypothesis of equality across categories was tested by the Kruskal-Wallis test. Correlation coefficients were computed between observed individual values of ΔFEV_1 _and the outcome, and between the category midpoint values of ΔFEV_1 _(-275 ml, 0 ml, 100 ml, 200 ml and 375 ml) and the category mean response of the outcome.

#### Model-based analyses

In line with established statistical procedures, generalised linear modelling [23,24] was performed to examine the relationship between ΔFEV_1 _and each outcome variable. For TDI and ΔSGRQ, observations at all timepoints were modelled together using repeated-measures multiple regression analyses, assuming constant variance and an unstructured correlation matrix. Time was included both as a main effect and in an interaction with ΔFEV_1_.

Rescue medication use and exacerbations were modelled as number of puffs and number of exacerbations, respectively, during time on treatment. Rescue medication use was modelled using the zero-inflated negative binomial distribution for likelihood-based model building, and then the final model was refitted using quasi-likelihood to report parameter estimates. Exacerbations were modelled using the negative binomial distribution. For both, in order to ensure positivity of the modelled mean response, the logarithm of the mean was represented as linear in the covariates, and then the mean was found by taking antilogs.

Other predictor variables were baseline trough FEV_1 _(continuous), age (continuous), gender (binary), ICS use (binary: yes or no), treatment (indacaterol, formoterol, tiotropium or placebo), screening FEV_1 _measured to assess reversibility before and after a short-acting β_2_-agonist, and before and after a short-acting anticholinergic, world region (Western Europe and the USA, Eastern Europe and Turkey, Rest-of-World), and time at risk for exacerbations and rescue medication use. Disease severity was included as a binary variable, based on the Global initiative for chronic Obstructive Lung Disease (GOLD) stages [19]; predominantly GOLD 2 (moderate or less severity including 91% moderate, referred to subsequently as GOLD 2) versus predominantly GOLD 3 (severe or greater severity including 98% severe, referred to as GOLD 3), as measured by per cent predicted FEV_1 _at screening after short-acting β_2_-agonist. The default condition for all models was: baseline FEV_1 _of 1.3 l, age 65 years, gender male, GOLD 2, no ICS use, indacaterol treatment, screening FEV_1 _before (and after) reversibility testing of 1.3 l (1.5 l) and Western Europe/USA region. All statistical comparisons were made relative to this combination of covariates, and unless otherwise stated, these were the values of the parameters used for predictions by the models.

Model-based inference steps were performed to test for interactions between ΔFEV_1 _and the covariates treatment, disease severity, ICS use and world region. For this purpose, disease severity was represented jointly by baseline FEV_1_, the binary severity indicator defined above, Baseline Dyspnoea Index (BDI) for TDI and baseline SGRQ for ΔSGRQ. To allow for the possibility of differing relationships for negative versus positive values of ΔFEV_1_, a possible breakpoint at ΔFEV_1 _= 0 was tested in each model. The main effects of covariates were tested for significance according to Wald P values in the final model, with P < 0.01 judged significant without any adjustments for multiplicity.

For each outcome variable, the improvement in expected response for an increase in FEV_1 _from 0 to 100 ml was also computed, based on a model that excluded treatment effects, to allow for variation in ΔFEV_1 _between, as well as within, treatments.

## Results

In total, 3313 patients were included in the analysis. Patient demographic and clinical characteristics are presented in Table 1. Age, pre- and post-bronchodilator FEV_1 _and body mass index were well balanced across studies. Study 1 included more males, patients taking ICS and patients with slightly lower per cent predicted FEV_1 _and reversibility.

**Table 1:** Baseline characteristics of study participants included in the analysis

	**Study 1 INVOLVE **[16]	**Study 2 INHANCE **[17]	**Study 3 INLIGHT 1 **[18]	Total
n	1377	1575	361	3313
Age, years	64 (8)	64 (9)	63 (10)	64 (9)
Male/female, %	78/22	63/37	52/48	69/31
Body mass index, kg/m^2^	27 (5)	27 (6)	28 (7)	27 (6)
FEV_1_, % predicted*	53 (14)	56 (14)	55 (14)	55 (14)
FEV_1_/FVC, %*	51 (10)	53 (10)	53 (10)	52 (10)
Pre-bronchodilator FEV_1_, l	1.35 (0.43)	1.33 (0.49)	1.34 (0.51)	1.34 (0.47)
Post-bronchodilator FEV_1_, l*	1.52 (0.47)	1.50 (0.50)	1.51 (0.52)	1.51 (0.49)
Reversibility, %*	13.2 (13.4)	15.5 (15.9)	16.0 (18.7)	14.6 (15.3)
ICS use yes/no, %	55/45	38/62	32/68	45/55
Smoker/ex-smoker, %	40/60	44/56	52/48	43/57
BDI score	6.6 (2.2)	6.5 (2.3)	NA	6.5 (2.2)
SGRQ total score	44 (18)	45 (18)	49 (19)	45 (18)
Treatments				
Placebo, n	322	311	176	809
Indacaterol 75 μg, n	0	67	0	67
Indacaterol 150 μg, n	0	346	185	531
Indacaterol 300 μg, n	363	357	0	720
Indacaterol 600 μg, n	344	68	0	412
Formoterol 12 μg, n	348	75	0	423
Tiotropium 18 μg, n	0	351	0	351

Data are mean (standard deviation) unless otherwise stated. *Measured 30 min after salbutamol 400 μg inhalation. Reversibility was calculated as the difference between the pre- and post-bronchodilator values of FEV_1 _(in l) as a percentage of the pre-bronchodilator value; BDI, Baseline Dyspnoea Index; FEV_1_, forced expiratory volume in 1 second; FVC, forced vital capacity; ICS, inhaled corticosteroid; INVOLVE, INdacaterol: Value in COPD: Longer term Validation of Efficacy and safety; INHANCE, INdacaterol versus tiotropium to Help Achieve New COPD treatment Excellence; INLIGHT, INdacaterol: efficacy evaLuation usInG 150 μg doses with COPD PatienTs; NA, not available; SGRQ, St George's Respiratory Questionnaire.

### Data summaries and related inferences

The distribution of average ΔFEV_1 _responses by timepoint is shown in Table 2, both as frequencies within ΔFEV_1 _categories and as percentiles of distributions. Median values ranged from 75 to 94 ml. Approximately 5% of observations were excluded due to extreme ΔFEV_1 _(± 500 ml) at any timepoint; 0.7% observations (24/3313) were less than -500 ml and 4.1% (137/3313) were greater than 500 ml at Week 12.

**Table 2:** Summary information for averages of ΔFEV_1_

		Number of observations in intervals defined by ml ranges							Percentiles* of observations						
**Average of ΔFEV**_**1**_	**n**	**< -500**	**-500, -50**	**-50, 50**	**50, 150**	**150, 250**	**250, 500**	**> 500**	**Minimum**	**5%**	**25%**	**50%**	**75%**	**95%**	**Maximum**

Week 4-12	3313	24	623	695	717	563	554	137	-1180	-198	-20	94	220	466	1966
Week 4-24/26	2389	16	478	476	550	388	377	104	-1148	-203	-30	88	214	474	1782
Week 4-52	1169	6	292	218	273	165	168	47	-755	-223	-52	75	201	464	1607
On treatment	3313	24	708	662	751	536	501	131	-1180	-213	-32	82	208	467	1966

*For example, when averaging over time on treatment, the minimum average ΔFEV_1 _was -1180 ml and the maximum was 1966 ml; between these points, 5% observations were less than or equal to -213 ml and half were less than or equal to 82 ml; ΔFEV_1_, change from baseline in trough forced expiratory volume in 1 second.

**Figure 1:**
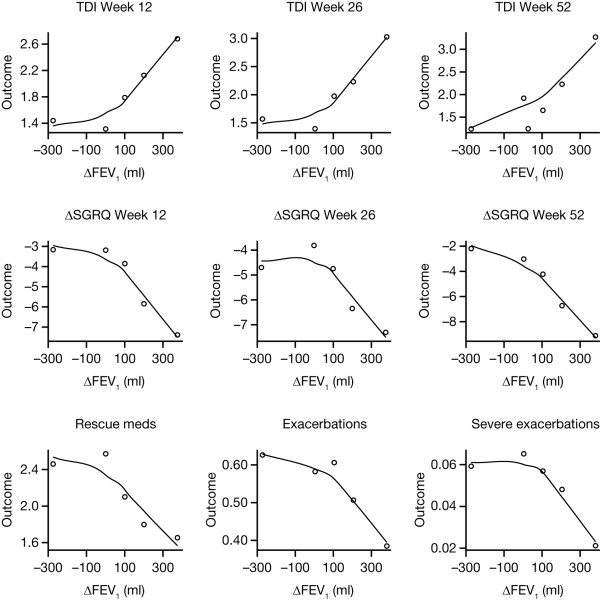
**Outcome means in ΔFEV_1 _categories versus category midpoint value**. Plots show data with Loess smooth curves superimposed. ΔFEV_1_, change from baseline in trough forced expiratory volume in 1 second; TDI, Transition Dyspnoea Index; ΔSGRQ, change from baseline in St George's Respiratory Questionnaire.

All relationships between ΔFEV_1 _and outcomes were statistically significant, except for severe exacerbations (Table 3). Individual-level correlations were weak (0.03-0.18), reflecting the large variability in outcomes; however, cohort-level correlations were stronger (0.79-0.95). When outcome means were plotted versus ΔFEV_1 _midpoints, there were clear trends towards greater improvement in outcomes with increasing ΔFEV_1_, particularly for positive ΔFEV_1 _(Figure 1). Responder rates, in terms of TDI and ΔSGRQ, followed a similar pattern to the mean outcomes (Table 4).

**Table 3:** Outcome means by average ΔFEV_1 _category, P values for associations between average ΔFEV_1 _and outcome, and correlations at individual and cohort levels

Average ΔFEV_1 _(ml)	Category midpoint value of ΔFEV_1 _(ml)	Withdrawal rate* (% patients)	TDI at 12 weeks (n = 2781)	TDI at 24/26 weeks (n = 2208)	TDI at 52 weeks (n = 1099)	ΔSGRQ at 12 weeks (n = 3141)	ΔSGRQ at 24/26 weeks (n = 2215)	ΔSGRQ at 52 weeks (n = 1115)	Rescue medication mean puffs per day (over study duration) (n = 3158)	Exacerbation rate (per year) (n = 3158)	Severe exacerbation rate (per year) (n = 3158)
-500, -50	-275	11.2	1.44	1.57	1.24	-3.15	-4.70	-2.21	2.46	0.63	0.059
-50, 50	0	9.0	1.31	1.39	1.92	-3.17	-3.81	-3.03	2.57	0.58	0.065
50, 150	100	10.1	1.79	1.97	1.65	-3.84	-4.74	-4.22	2.10	0.61	0.057
150, 250	200	10.2	2.12	2.23	2.23	-5.84	-6.34	-6.70	1.80	0.51	0.048
250, 500	375	6.7	2.68	3.03	3.27	-7.38	-7.29	-9.06	1.66	0.38	0.021

P value (Kruskal-Wallis)			< 0.001	< 0.001	< 0.001	< 0.001	< 0.001	< 0.001	< 0.001	< 0.001	0.1

Correlation, individual level			0.15	0.14	0.18	-0.12	-0.07	-0.16	-0.11	-0.06	-0.03

Correlation, cohort level			0.90	0.88	0.92	-0.90	-0.79	-0.95	-0.88	-0.89	-0.81

*Non-completers in all studies categorised by ΔFEV_1 _at Day 2; ΔFEV_1_, change from baseline in trough forced expiratory volume in 1 second; TDI, Transition Dyspnoea Index; ΔSGRQ, change from baseline in St George's Respiratory Questionnaire

**Table 4:** Responder rates* for TDI^† ^and **Δ**SGRQ^† ^by average **Δ**FEV_1 _category

Average ΔFEV_1 _(ml)	Category midpoint value of ΔFEV_1 _(ml)	TDI at 12 weeks % responders (n = 2781)	TDI at 24/26 weeks % responders (n = 2208)	TDI at 52 weeks % responders (n = 1099)	ΔSGRQ at 12 weeks % responders (n = 3141)	ΔSGRQ at 24/26 weeks % responders (n = 2215)	ΔSGRQ at 52 weeks % responders (n = 1115)
-500, -50	-275	50	51	45	42	49	41
-50, 50	0	48	49	53	46	45	45
50, 150	100	54	57	50	48	48	49
150, 250	200	59	60	58	53	59	54
250, 500	375	66	69	69	56	57	65

*Responders were patients who achieved at least the minimal clinically important difference (MCID; one and four units for TDI and ΔSGRQ, respectively); TDI, Transition Dyspnoea Index; ΔSGRQ, change from baseline in St George's Respiratory Questionnaire; ΔFEV_1_, change from baseline in trough forced expiratory volume in 1 second.

^†^For the outcomes, each row contains approximately 20% of the column's indicated n, as in the left half of Table 2

### Model-based results

**Figure 2:**
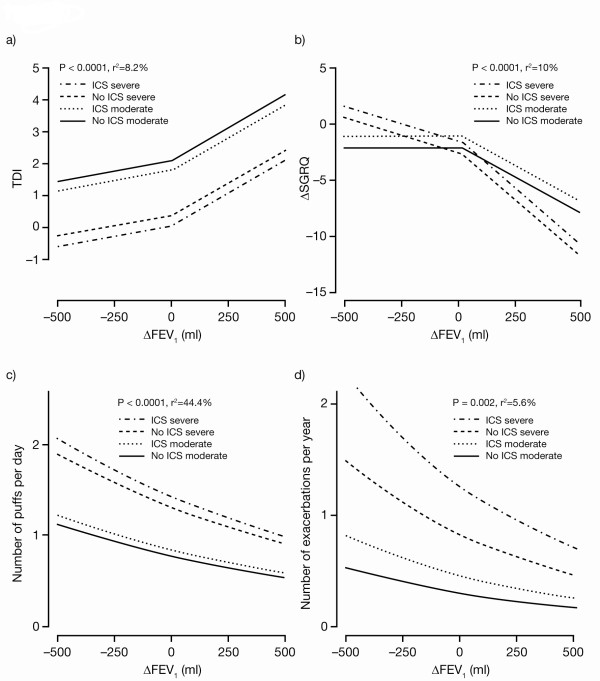
**Outcomes versus ΔFEV_1_: curves fitted from model-based analysis**. SGRQ and TDI data is for Week 24/26. Exacerbations are reported counts normalised to 1 year. Rescue medication use is reported numbers of puffs normalised to 1 day. For plotting predicted curves, 'moderate' refers to baseline FEV_1 _1.595 l (third quartile of observed values) and GOLD 2 (moderate or less [19]); 'severe' refers to baseline FEV_1 _0.95 l (first quartile of observed values) and GOLD 3 (severe or greater [19]). For TDI, 'moderate' and 'severe' refer to BDI at quartiles 2 and 1, respectively. For ΔSGRQ, 'moderate' and 'severe' refer to baseline SGRQ at quartiles 31.3 and 58.13, respectively; ΔFEV_1_, change from baseline in trough forced expiratory volume in 1 second; ΔSGRQ, change from baseline in St George's Respiratory Questionnaire; TDI, Transition Dyspnoea Index; GOLD, Global initiative for chronic Obstructive Lung Disease; BDI, Baseline Dyspnoea Index; ICS, inhaled corticosteroid.

The plots of curves fitted from the model-based analysis for each outcome variable versus ΔFEV_1 _are presented in Figures 2a-d. For TDI and ΔSGRQ, the significant breakpoints at zero are evident in the changes of slope in the fitted lines. For rescue medication and exacerbations the breakpoints were not significant. The fitted curves for rescue medication and exacerbations are linear on logarithmic scales, so appear nonlinear on the scales of these plots.

ΔFEV_1 _was significantly correlated with TDI score (P < 0.0001). A significant breakpoint in the fitted lines is seen at zero; the slope was significantly shallower for negative ΔFEV_1 _compared with positive ΔFEV_1 _(P = 0.003 for the difference between slopes). The slope of the relationship (determining the magnitude of change in outcome for a given improvement in ΔFEV_1_) was not significantly affected by treatment, baseline severity, ICS use or world region. Hence, the overall model-predicted increase in TDI for a 100 ml increase in ΔFEV_1 _was the same for all combinations of covariates, and estimated to be 0.46 at Week 24/26. Although the slope of the relationship with ΔFEV_1 _was the same for all covariates, the intercept, that is, the TDI corresponding to zero change in FEV_1_, was not. For a given ΔFEV_1_, patients with lower baseline FEV_1_, lower BDI, using ICS or on placebo, had significantly lower values of TDI, while those from Eastern Europe/Turkey and Rest-of-World regions had significantly higher values. When covariates representative of patients who were less severe were inputted into the model (i.e., GOLD 2, no ICS and baseline FEV_1 _of 1.595 l), the model-predicted TDI for a zero and +100 ml change in FEV_1 _was 1.98 and 2.44, respectively. For more severe patients (i.e., GOLD 3, ICS and baseline FEV_1 _of 0.95 l), the model-predicted TDI for patients with zero and +100 ml change in FEV_1 _was -0.20 and 0.26, respectively.

There was a significant correlation between ΔSGRQ and ΔFEV_1 _(P < 0.0001). As with TDI, the slope of the relationship between ΔSGRQ and ΔFEV_1 _was significantly shallower for negative ΔFEV_1 _(P = 0.002 for the difference between slopes). The slope of the relationship with improvement in FEV_1 _was not significantly affected by treatment, ICS use, or world region, but it was steeper for patients in GOLD 3, and with baseline FEV_1 _0.95 l compared with GOLD 2 and baseline FEV_1 _1.595 l (P = 0.004). For an increase of ΔFEV_1 _of 100 ml, the model predicted a change in SGRQ of -1.3 for GOLD 2 and -1.9 for GOLD 3 patients at Week 24/26. Patients with worse baseline FEV_1_, with worse baseline SGRQ, using ICS or on placebo, had significantly higher ΔSGRQ, whereas patients from Eastern Europe/Turkey and Rest-of-World regions had significantly lower ΔSGRQ at Week 24/26. For GOLD 2 patients, who had used no ICS and had baseline SGRQ of < 31, the model-predicted improvement in SGRQ at Week 26 for a zero and +100 ml change in FEV_1 _was -1.6 and -2.9, respectively. Similarly, for GOLD 3 patients who had used ICS and had baseline SGRQ of > 58, the model-predicted improvement in SGRQ at Week 24/26 was -0.9 and -2.8, respectively.

ΔFEV_1 _was significantly correlated with rescue medication use (P < 0.0001). Treatment, baseline severity, ICS use or world region, did not significantly affect the slope of the relationship, and the slope did not change significantly between negative and positive ΔFEV_1_. Hence, for all combinations of covariates, an increase of 100 ml in ΔFEV_1 _is predicted to yield the same 10% reduction in rescue medication use. Patients with lower baseline FEV_1_, male patients, those with higher baseline medication usage or more severe disease, using ICS or on placebo or tiotropium, had significantly higher rates of rescue medication usage. Younger patients (< 65 years) had almost significantly higher rates (P = 0.012, versus the defined significance level of P < 0.01). For GOLD 2 patients not receiving ICS, the predicted daily number of puffs of rescue medication for a zero and +100 ml ΔFEV_1 _was 0.89 and 0.80, respectively, and 1.83 and 1.64 for those in GOLD 3 and using ICS.

ΔFEV_1 _was significantly correlated with exacerbations (P = 0.002). Treatment, baseline severity, ICS use or world region, did not significantly affect the slope of the relationship. Furthermore, the slope did not change significantly between negative and positive ΔFEV_1_. Hence, for all combinations of covariates, an increase of 100 ml in ΔFEV_1 _is predicted to yield the same 12% decrease in exacerbations. Patients with lower baseline FEV_1 _and patients using ICS had significantly higher rates of exacerbations. Patients from the Eastern Europe/Turkey region had significantly lower rates of exacerbations. The model estimate for the annual rate of exacerbations for patients with a zero and +100 ml ΔFEV_1 _were 0.29 and 0.25, respectively, for GOLD 2 patients not using ICS; and 1.28 and 1.12, respectively, for patients in GOLD 3 and using ICS. As in the summary analysis, the rate of severe exacerbations requiring hospitalisation was not significantly correlated with ΔFEV_1 _(P = 0.3).

## Discussion

Our analyses show that improvement in FEV_1 _is significantly related to changes in the patient-reported outcomes TDI, SGRQ, exacerbation rate and rescue medication use over 12-52 weeks of treatment. These relationships were significant at both an individual and population level, although correlations were much stronger in the population-based analyses.

Few studies have examined the relationship between change in FEV_1 _and change in outcomes. However, our results are consistent with analyses of patients from the 3-year EUROSCOP (The European Respiratory Society Study on Chronic Obstructive Pulmonary disease) study, in which an improvement of 100 ml in FEV_1 _was associated with a 4% reduction in dyspnoea in males [13], and a 16-week clinical study, in which a significant, but weak correlation between change in FEV_1 _and change in SGRQ score was demonstrated (r = 0.33, P = 0.001) [14]. Further, a recent systematic review of 22 studies found that 100 ml increase in FEV_1 _was associated with a statistically significant reduction in SGRQ of 2.5 [15]. However, to our knowledge, the current analysis is the largest and most comprehensive to investigate the correlation between change in FEV_1 _and change in outcomes using individual patient data from studies of similar design. This provides a relatively homogeneous population for analysis, compared with study-level meta-analyses.

We demonstrated that a 100 ml increase in trough FEV_1 _(a magnitude of change with perceptible effects [25]) was associated with a 0.46-unit increase in TDI and a 1.3- to 1.9-unit improvement in SGRQ after a 24/26-week treatment period and, over 12-52 weeks of treatment, a 10% decrease in daily rescue medication use and a 12% decrease in the annual exacerbation rate. In general, we found that treatment, baseline severity, concomitant ICS use and world region, did not affect the slope of the relationship between outcome and change in FEV_1_, except for ΔSGRQ where more severe COPD, as characterised by a lower FEV_1 _and a higher SGRQ at baseline, was associated with a steeper slope, compared with less severe COPD. This is consistent with results from the 3-year TORCH (TOwards a Revolution in COPD Health) study, in which trends to greater improvement in SGRQ with worsening GOLD severity were noted with active treatments [26].

Although severe exacerbations showed a trend toward greater reductions with increasing ΔFEV_1_, the relationship was not statistically significant. While the observed 12% reduction in overall exacerbation rate for an improvement of 100 ml in FEV_1 _was comparable with previously published data [11], the studies included in our analysis were not powered to show an effect on exacerbations, and did not specifically recruit patients at risk of exacerbations.

We found inconsistent effects of different treatments across individual outcomes, perhaps due to patient numbers in sub-categories being too low to demonstrate consistent differences for individual treatments across all outcomes. However, our analysis did demonstrate that the relationship between ΔFEV_1 _and outcome appeared to be the same, regardless of treatment arm. Similarly, baseline severity, ICS use and world region were assessed as main effects, as well as for their potential influence on the effect of ΔFEV_1_. Although numbers of patients in GOLD 4 (as well as GOLD 1) were too small to make any inferences, patients predominantly in GOLD 3 at baseline, and those using ICS, consistently exhibited significantly worse outcomes. Indeed, the variability in baseline severity and ICS use are likely to have been major contributors to the large variability in observed outcomes.

The relationships between outcomes and ΔFEV_1 _may differ between negative and positive ΔFEV_1_, and for this reason, the models included a possible breakpoint at zero in the relationship slope. The inclusion of this breakpoint was found to be significant for TDI and ΔSGRQ, suggesting that baseline severity and other included covariates could not explain the observed behaviour fully. These results may have been influenced by differences in withdrawal rates between categories [27], since the highest withdrawal rate was in those with a negative change in FEV_1_, although differences between groups were minimal. The inclusion of a breakpoint was not significant for rescue medication and exacerbations, even though Figure 1 may have anticipated its importance, especially for exacerbations. The large variability and count nature of the data for rescue medication and exacerbations may have caused 'Type-2' statistical errors, i.e., failure to find the true breakpoints to be significant.

We found that zero change in FEV_1 _was associated with significant positive improvements in TDI and SGRQ. Additionally, while a greater proportion of patients achieved the MCID for TDI and SGRQ as ΔFEV_1 _increased, our results indicated that as many as 50% patients responded, irrespective of ΔFEV_1_, possibly an effect of clinical trial participation seen consistently in the placebo limb of clinical trials [28-30].

We constructed the models in our analysis using ΔFEV_1 _as a predictor, and the other outcome measures as the response variables, based on the results of a carefully-controlled series of clinical trials. However, ΔFEV_1 _was as much a response as was the outcome, so ΔFEV_1 _was not an 'independent' variable controlled as part of the experimental design. There may have been further confounders that simultaneously affected how both ΔFEV_1 _and the outcome responded to treatment. The fitted models therefore describe the observed relationships under the conditions of a clinical trial, but do not provide a definitive answer as to whether there is a causal relationship between ΔFEV_1 _and the outcomes.

The studies included in our analysis were powered on the spirometric endpoint FEV_1_, which is required by regulatory agencies for the approval of bronchodilators, and is included in the majority of treatment guidelines. For this reason we made FEV_1 _the focus of our analysis. Other physiological parameters such as inspiratory capacity may have stronger correlations with dyspnoea [31]. However data for these parameters were not available from our dataset and further research is needed to investigate such correlations in large numbers of patients.

## Conclusions

It is commonly stated that spirometry does not fully capture the impact of COPD on a patient's health [32]. Our analysis of a large cohort of patients has demonstrated that in individual subjects, change in FEV_1 _is a significant, albeit relatively weak predictor of improvement in patient-reported outcomes. However, the current analysis also shows that, at a population level, improvements in FEV_1 _with long-acting bronchodilator therapy are strongly correlated with improvements in dyspnoea, health status and exacerbations. This suggests that interventions which significantly improve FEV_1 _are also likely to be associated with improved clinical and patient-reported outcomes.

## List of abbreviations

BDI: Baseline Dyspnoea Index; COPD: Chronic Obstructive Pulmonary Disease; EUROSCOP: The European Respiratory Society Study on Chronic Obstructive Pulmonary disease; FEV_1_: Forced Expiratory Volume in 1 second; FVC: Forced Vital Capacity; GOLD: Global initiative for chronic Obstructive Lung Disease; ICS: Inhaled Corticosteroid; INHANCE: INdacaterol versus tiotropium to Help Achieve New COPD treatment Excellence; INLIGHT: INdacaterol: efficacy evaLuation usInG 150 μg doses with COPD PatienTs; INVOLVE: INdacaterol: Value in COPD: Longer term Validation of Efficacy and safety; MCID: Minimal Clinically Important Difference; SGRQ: St. George's Respiratory Questionnaire; TDI: Transition Dyspnoea Index; TORCH: TOwards a Revolution in COPD Health.

## Competing interests

PWJ has received consultancy fees and honoraria from AstraZeneca, Almirall, Bayer, Boehringer Ingelheim, Chiesi, GlaxoSmithKline, Novartis, Pfizer, Roche and Spiration; and grants for his institution from GlaxoSmithKline and Novartis.

JFD has received consultancy fees and honoraria from Novartis, Almirall, Forest laboratories, Boehringer Ingelheim, GlaxosSmithKline, Pfizer, Sunovion Dey and Talecris.

JN, GP and CL are employees of Novartis. SP is an ex-employee of Novartis.

## Authors' contributions

PWJ participated in the design and analysis planning and advised on the interpretation of the study. JFD participated in the design and analysis planning and advised on the interpretation of the study. JN developed the design, concept of the study and analysis, and carried out the statistical analysis. SP conceived of the study, participated in its design and analysis planning and contributed to its interpretation. GP programmed the analysis data set. CL conceived of the study, participated in its design and analysis planning and contributed to its interpretation.

All authors had full access to the data and were involved in drafting the manuscript. All authors read and approved the final manuscript.
